# Utility of serum and urine leucine-rich alpha-2 glycoprotein 1 (LRG1) as predictors of appendicitis and complicated appendicitis in children

**DOI:** 10.1007/s00383-025-06008-8

**Published:** 2025-04-10

**Authors:** Johanna Gudjonsdottir, Bodil Roth, Bodil Ohlsson, Lars Hagander, Martin Salö

**Affiliations:** 1 Department of Clinical Sciences in Lund, Pediatrics, Lund University, Lasarettsgatan 48, 221 85 Lund, Sweden; 2 Department of Surgery, Skåne University Hospital, Malmö, Sweden; 3 Department of Internal Medicine, Skåne University Hospital, Malmö, Sweden; 4 Department of Clinical Sciences, Lund University, Malmö, Sweden; 5 Department of Pediatric Surgery, Skåne University Hospital, Lund, Sweden

**Keywords:** LRG1, Appendicitis, Pediatric

## Abstract

**Purpose:**

Leucine rich alpha-2 glycoprotein 1 (LRG1) has emerged as a promising biomarker for appendicitis, especially in pediatric patients. However, the currently available data are sparse, and the biomarker must be validated in more settings and compared to standard inflammatory markers. We aimed to evaluate the diagnostic and discriminative utility of serum and urine LRG1 in children with other causes of abdominal pain (no appendicitis) versus appendicitis, and uncomplicated versus complicated appendicitis.

**Methods:**

The study design was prospective including children ≤ 15 years with suspected appendicitis. Blood and urine samples were collected at the time of clinical evaluation at the Pediatric Emergency Department and analyzed for concentrations of LRG1. Appendicitis diagnosis and severity were determined through histopathological examination and intraoperative findings. Group comparisons were carried out using Kruskal–Wallis test with post hoc Dunn–Bonferroni tests for pairwise comparisons. Associations between LRG1 and other laboratory and clinical variables and the odds of appendicitis and complicated appendicitis were assessed by univariate and multivariable logistic regression analyses. Diagnostic (no appendicitis versus appendicitis) and discriminative (uncomplicated versus complicated appendicitis) performance were evaluated through Receiver Operating Characteristic (ROC) curves with analyses of Areas Under the Curve (AUC). Optimal cutoffs were generated using Youden’s index, and diagnostic and predictive values were calculated and compared.

**Results:**

172 children were included. 132 (77%) had appendicitis and 56 (42%) of these had complicated appendicitis. The median age was 10 (IQR 8–12) years and 98 (57%) were boys. Serum concentrations of LRG1 did not differ significantly between the groups. Urine LRG1 was significantly higher among children with complicated appendicitis and no appendicitis compared to children with uncomplicated appendicitis (p < 0.001). In the logistic regression analysis of all children with suspected appendicitis, increased serum LRG1 was associated with a decreased odds of appendicitis (OR 0.96 [95% CI 0.93–0.99], p = 0.008). This association remained after adjustment for age, sex and symptom duration (aOR 0.95 [0.92–0.98], p = 0.003). Urine LRG1 was not associated with the odds of appendicitis. Neither serum nor urine LRG 1 were significantly associated with the odds of complicated appendicitis. When it comes to diagnosing appendicitis, both serum and urine LRG1 had AUC values of 0.39. However, urine LRG had a specificity of 95% and a PPV of 83%. The discriminative performance of serum LRG1 was poor, but the AUC for urine LRG1 of 0.65 was better than the ones for leukocytes, neutrophils and neutrophil percentages. Still, it was lower than the AUCs for C-reactive protein (CRP) and Appendicitis Inflammatory Response (AIR) score. Urine LRG1 has a high specificity and PPV for all cases of appendicitis, and correctly identifies cases of complicated appendicitis to a greater extent than some of the currently available inflammatory markers. Still, the regression analyses show no significant associations between urine LRG1 and appendicitis and complicated appendicitis in children.

**Conclusion:**

In contrast to previous studies, in this cohort serum LRG1 was associated with decreased odds of appendicitis, shedding some doubt over the clinical utilization of serum LRG1 as a biomarker for appendicitis in children.

## Introduction

Appendectomy is the most common acute abdominal operation worldwide [1]. The lifetime risk of appendicitis is 7–9%, and the incidence peaks during the second decade of life [2]. Despite being such a common condition, obtaining a correct diagnosis might be a challenge, especially in younger children [3, 4]. Among all children evaluated at pediatric emergency departments due to abdominal pain, 4–9% are diagnosed with appendicitis [5–7], and safely ruling out or confirming the diagnosis is of high clinical value. Although many cases of appendicitis heal spontaneously [8], still most children are treated surgically. However, in the not so far future, non-surgical treatment of uncomplicated appendicitis [9] might be standard clinical practice. As a consequence, the requirements of the diagnostic aids for appendicitis diverge; not only should they be able to help clinicians distinguish appendicitis from other causes of acute abdominal pain, but they should also distinguish uncomplicated from complicated appendicitis.

Among other diagnostic aids, novel biomarkers for appendicitis are being evaluated. During recent years, leucine-rich alpha-2 glycoprotein 1 (LRG1) has emerged as a potentially promising predictor of both pediatric appendicitis and complicated appendicitis [10]. The protein is involved in signal transduction and pathogenesis of a variety of diseases in several different organ systems [11]. In the appendicitis context, LRG1 has been analyzed in serum, urine, and saliva, with varying – but sometimes very promising—diagnostic accuracy, especially for urine LRG1 [12]. When analyzed in urine samples, LRG1 is a non-invasive biomarker, giving it a potential advantage over currently available inflammatory markers, which normally require blood sampling. However, the currently available research on the topic is still quite sparse [12], and as with all new clinical predictors, their performance must be validated in different settings and study populations. Furthermore, only a few previous studies have compared the diagnostic utility of LRG1 to other commonly used inflammatory markers such as C-reactive protein (CRP), leukocytes and neutrophils [10, 13–16].

### Aim

To evaluate the association of serum and urine LRG1 concentrations with the odds of appendicitis and complicated appendicitis, and to assess the biomarkers’ diagnostic and discriminative performance compared to other commonly used inflammatory markers.

## Materials and methods

### Study design

A prospective cohort study with patient enrollment was conducted from December 2017 to February 2021. Children aged 15 years or younger were included at the Pediatric Emergency Department at Skåne University Hospital, Lund, Sweden—a tertiary pediatric hospital with an uptake area of 350,000 inhabitants for general surgical emergencies. The study was approved by the regional ethics committee (Regionala Etikprövningsmyndigheten, Lund, Sweden, DNR 2013/614) and the hospital review board (Skåne University Hospital, Lund, Sweden). All included subjects agreed to participation through informed parental written consent. All research was performed in accordance with relevant guidelines and regulations.

### Inclusion and exclusion criteria

All pediatric patients referred to the on-call pediatric surgeon for evaluation due to suspected appendicitis (defined as acute pain in the right lower quadrant) were eligible for inclusion. Exclusion criteria were previous episode of suspected or confirmed appendicitis, severe chronic illness, or ongoing treatment with anti-Inflammatory drugs including inhalation corticosteroids, in order to avoid conditions and treatments which might affect the clinical and/or laboratory presentation.

### Data collection

Data was primarily collected at the time of evaluation at the Pediatric Emergency Department. The on-call pediatric surgeon responsible for the assessment of the patient, registered the following information in a study protocol: current symptoms and symptom duration, findings on clinical examination and results of standard laboratory tests, including C-reactive protein (CRP), leukocytes and neutrophils. The medical records of the included patients were later reviewed to obtain information regarding the presence of an appendicolith (diagnosed perioperatively or on Ultrasonography (US) or Computed Tomography (CT)), results from histopathological examinations of the appendix specimens and final diagnoses. Based on clinical and laboratory results, Appendicitis Inflammatory Response (AIR) scores were calculated for each patient. AIR score is a clinical prediction model for appendicitis that was originally developed on and for adult patients but has been validated on children and shown to outperform both the more well-known Pediatric Appendicitis Score (PAS) and Alvarado score [17].

### Definitions

All study participants were allocated to one of three groups, based on their final diagnoses: no appendicitis, uncomplicated appendicitis and complicated appendicitis. Appendicitis diagnosis was based on intraoperative findings and histopathological examination. Uncomplicated appendicitis was phlegmonous appendicitis, histopathologically defined as an infiltration of polymorphonuclear granulocytes in the muscularis propria layer. Complicated appendicitis was defined as gangrenous or perforated appendicitis, and appendiceal abscess. Gangrenous appendicitis was defined as a full-thickness necrosis of the appendiceal wall. Perforated appendicitis was defined as a visible hole in the appendix, or a finding of an appendicolith or free pus in the abdominal cavity.

### Analyses of routine blood tests

C-reactive protein (CRP), leukocytes and neutrophils were analyzed according to standard protocol at the Department of Clinical Chemistry, Skåne University Hospital, Lund, Sweden. Neutrophil percentages were calculated by dividing the number of neutrophils with the total number of leukocytes.

### Analyses of urine and serum LRG1

After sampling, blood and serum tubes were put in an upright position for 30 min before centrifugation at 2000G and 1300G for blood and urine, respectively. Each sample was then allocated to one to three separate tubes of 0.5 mL, depending on the amount of serum/urine available, and frozen to – 80 °C. The frozen samples were stored at a regional biobank until analyzed with a competitive inhibition ELISA kit (Cusabio, Hubei Province, China, CSB-E12962 h) according to the manufacturer’s manual. Standards (1, 0.1, 0.4, 2.0, 10, and 40 μg/mL), serum and urine samples (50 μL/well), and horseradish peroxidase (HRP) conjugated LRG1 were pipetted in duplicate into a plate pre-coated with anti-LRG1 monoclonal antibody. The unbound conjugate was washed off and substrate solutions were added. The reaction was stopped, and the absorbance was measured at 450 nm. The amounts of HRP conjugate were inversely proportional to the concentrations of LRG1 in the samples, and each LRG1 concentration was interpolated from the standard curve. Intra- and inter assay coefficients of variation (CV) were < 12% and < 15%, respectively.

### Statistical analyses

Continuous non-normally distributed data are presented as median and interquartile range (IQR), with group differences assessed using the Kruskal–Wallis test with posthoc Dunn–Bonferoni tests for pairwise comparisons. Categorical data are presented as absolute numbers and percentages (%), with group comparisons using either the Fisher’s exact test or the Chi-squared test. Associations between independent variables and different outcome variables (appendicitis and complicated appendicitis) were assessed through univariate and multivariable logistic regression analyses, with results presented as odds ratios (ORs) with 95% confidence intervals (CIs). Independent variables thought to function as confounding factors (age, sex and symptom duration) were included in the multivariable analyses. ROC analyses including Area Under the Curve (AUC) values were used to compare the diagnostic performances of urine and serum LRG1 with currently available biomarkers as well as Appendicitis Inflammatory Response (AIR) score. Based on the ROC curves, the cut-off values maximizing both test sensitivity and specificity were identified using Youden’s index. Based on these cut-offs positive and negative predictive values (PPVs and NPVs) were calculated. For AIR score, previously suggested cutoffs for low (0–3 points) and high (9–12 points) risk were used [18, 19]. MedCalc online calculator (https://www.medcalc.org/calc/diagnostic_test.php) was used to generate 95% confidence intervals (CIs) for sensitivity, specificity, PPV and NPV. All other statistical analyses were performed in IBM SPSS statistics 29.0.

## Results

### Study population demographics

**Fig. 1:**
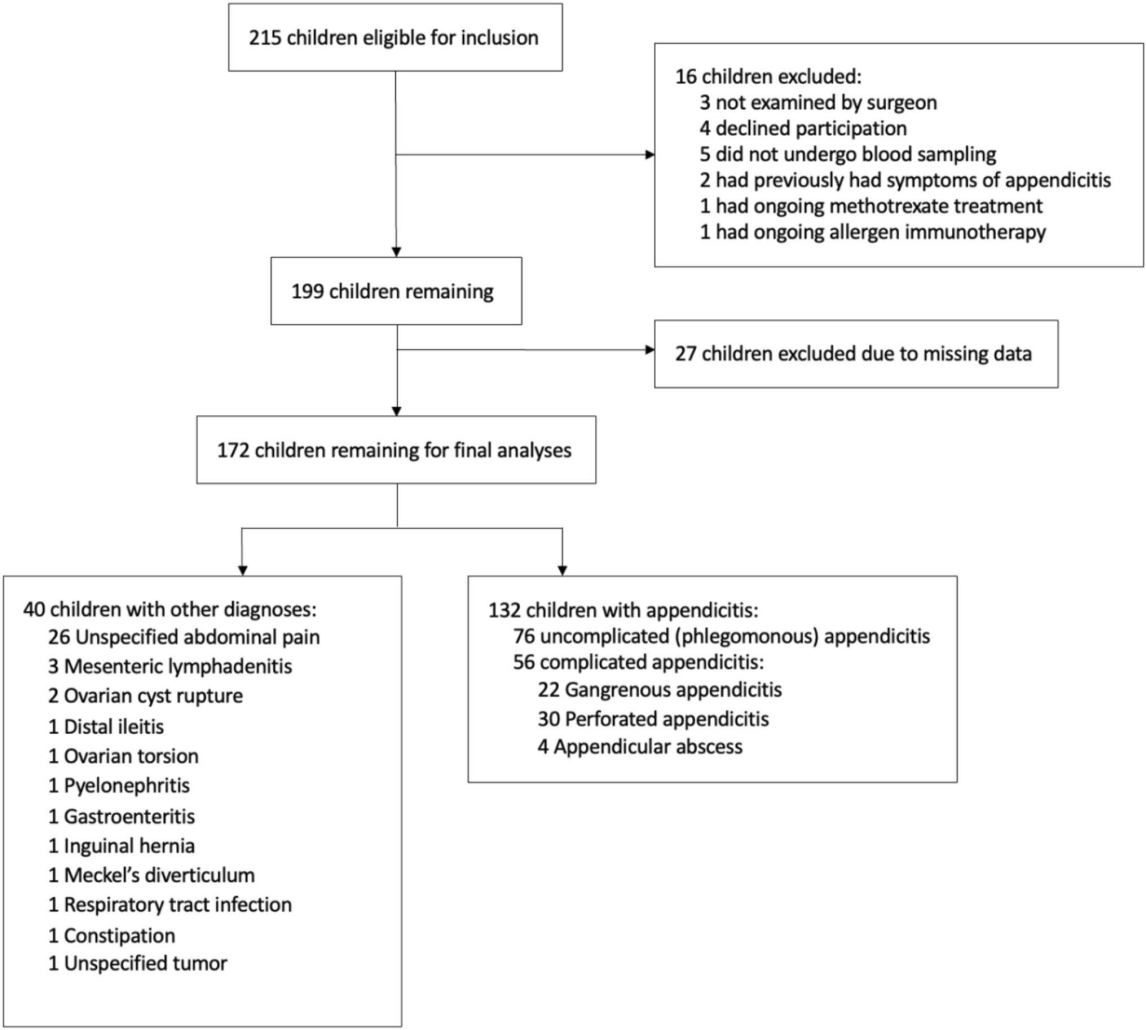
Flow chart of inclusion and exclusion of patients with suspected appendicitis

A total of 215 children were eligible for inclusion, and after exclusion of a total of 43 children, 172 remained for further analyses (Fig. 1). 132 (77%) had appendicitis, and of these, 56 (42%) had complicated appendicitis. All non-appendicitis diagnoses are presented in Fig. 1. In this group, 26 (65%) children had unspecified abdominal pain, meaning that their symptoms could not be explained by any certain pathology and that they received the diagnose code “R10.4” according to the International Classification of Diseases, 10 th version (ICD-10). The median age in the total cohort was 10 (IQR 8–12) years, and 99 (57%) were boys.

### Clinical and laboratory parameters among the different diagnose groups

When comparing the patients in the different diagnose groups, the children with complicated appendicitis were significantly younger (9 [IQR 7–11.5] years) than the ones with uncomplicated appendicitis (11 [IQR 9–13] years, p = 0.019) (Table 1). The distribution of symptom duration before presentation also varied among the groups, where a majority of patients (53%) with uncomplicated appendicitis presented within 24 h, while most patients (39%) with complicated appendicitis presented 24–28 h after onset of symptoms. Regarding standard inflammatory markers, the patients with both uncomplicated and complicated appendicitis had significantly higher concentrations of leukocytes and neutrophil percentages than the ones without appendicitis (p < 0.001 for both biomarkers). CRP concentrations were significantly higher among the patients with complicated appendicitis compared to the patients with no or uncomplicated appendicitis (p < 0.001). Appendicitis Inflammatory Response scores were significantly different in all pairwise comparisons: 4 (IQR 3–6) in the no appendicitis group, 6 (5–8) among the patients with uncomplicated appendicitis, and 8 (IQR 6.25–10) in the complicated appendicitis group (p < 0.001). The prevalence of an appendicolith was higher among the patients with complicated appendicitis (37%) compared to the ones with uncomplicated appendicitis (17%), p < 0.001.

**Table 1:** Demographics, symptom durations, inflammatory markers and serum and urine LRG1, AIR scores, and appendicolith statuses of 172 children with suspected appendicitis

	No appendicitis n = 40	Uncomplicated appendicitis n = 76	Complicated appendicitis n = 56	p value
Age (years)	11 (9–12.75)	11 (9–13)	9 (7–11.75)	**0.019**^**a**^
Sex (male)	16 (40%)	46 (61%)	36 (64%)	**0.043**
Symptom duration (h)				**0.023**
0–24	14 (35%)	40 (53%)	18 (32%)	
24–48	13 (33%)	27 (36%)	22 (39%)	
48–96	9 (23%)	8 (11%)	13 (23%)	
> 96	4 (10%)	0 (0%)	2 (4%)	
CRP (mg/L)	12 (5–41)	21 (8–39)	51 (27–121)	** < 0.001**^**b**^
Leukocytes (× 10^9^/L)	10 (6.7–14.8)	14.4 (11.8–18.0)	15.8 (13.0–19.0)	** < 0.001**^**c**^
Neutrophils (× 10^9^/L)	6.5 (4.2–10.6)	11.6 (9.3–14.7)	12.9 (10.3–15.8)	** < 0.001**^**c**^
Neutrophil percentage (%)	67 (57–78)	80 (75–85)	83 (76–86)	** < 0.001**^**c**^
AIR score	4 (3–6)	6 (5–8)	8 (6.25–10)	** < 0.001**^**d**^
Appendicolith	0 (0%)	13 (17%)	21 (37%)	** < 0.001**
Serum LRG1 (μg/mL)	27.6 (19.7–36.3)	24.8 (18.7–30.1)	23.4 (18.9–30.7)	0.124
Urine LRG1 (μg/mL)	0.33 (0.10–0.72)	0.10 (0.02–0.29)	0.29 (0.12–1.07)	** < 0.001**^**e**^

Values presented as median (IQR) and as absolute number and percentage of patients; n (%). Group differences were assessed through Kruskal–Wallis test with post hoc Dunn–Bonferroni tests for pairwise comparisons for continuous data and with Fisher’s exact test and Chi-squared test for categorical data. Symptom duration, CRP n = 40, 75 and 55, and leukocytes, neutrophils, and neutrophil percentage n = 39, 74 and 55, due to missing values

*CRP* C-reactive protein, *AIR score* Appendicitis Inflammatory Response score, *LRG1* Leucine-rich alpha-2 glycoprotein 1, *N/A* Not applicable

p < 0.05 was considered statistically significant (in bold)

^a^Significant when comparing uncomplicated appendicitis with complicated appendicitis,

^b^Significant when comparing no appendicitis with complicated appendicitis and uncomplicated appendicitis with complicated appendicitis,

^**c**^Significant when comparing no appendicitis with uncomplicated appendicitis and no appendicitis with complicated appendicitis,

^d^Significant in all pairwise comparisons,

^e^Significant when comparing no appendicitis with uncomplicated appendicitis and uncomplicated appendicitis with complicated appendicitis

### Groupwise comparisons of serum and urine LRG1 concentrations

**Fig. 2:**
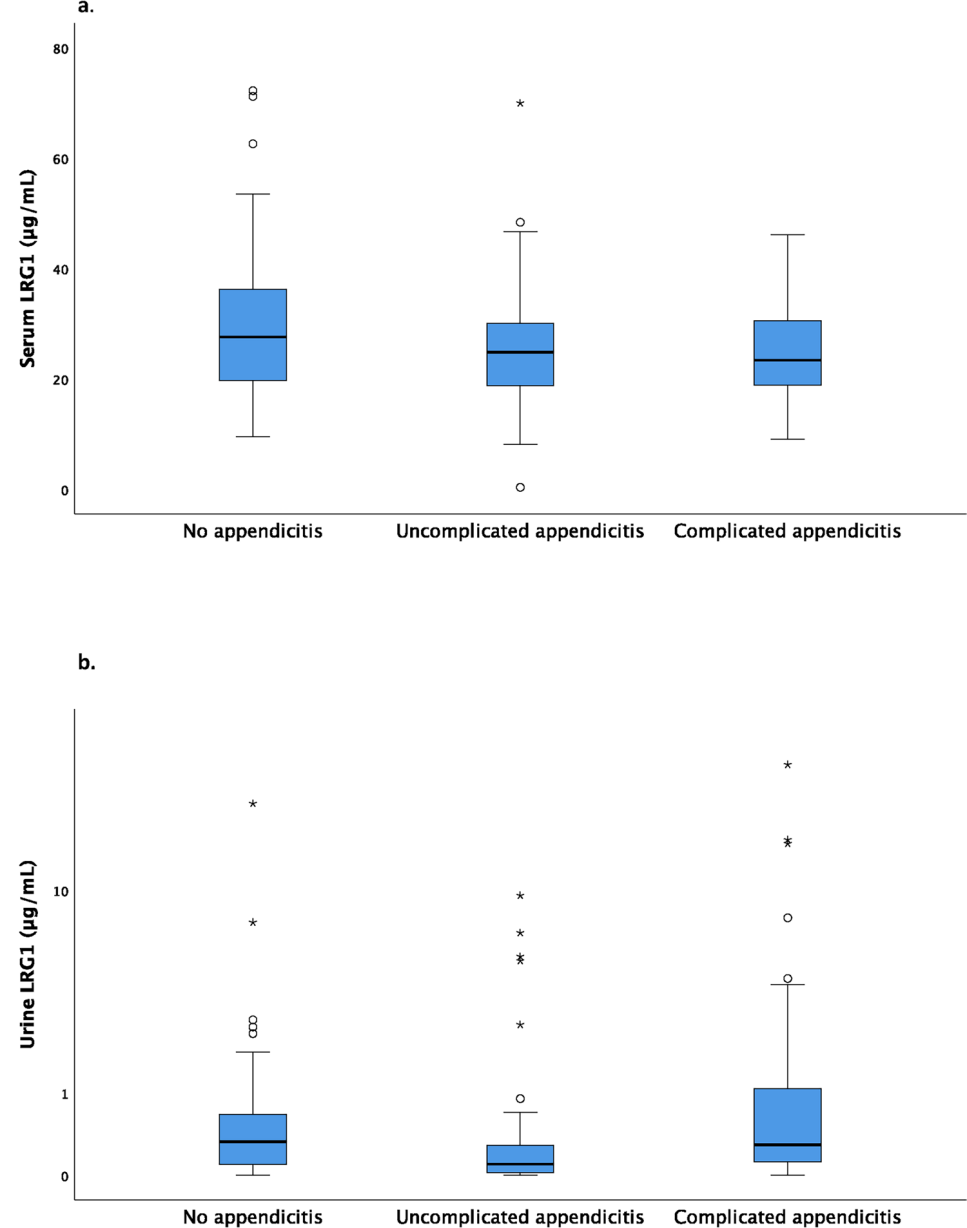
**a** Serum LRG1 in 172 children with suspected appendicitis. Group differences were assessed through Kruskal–Wallis test, p = 0.124. **b** Urine LRG1 in 172 children with suspected appendicitis. Group differences were assessed through Kruskal–Wallis test with post hoc Dunn–Bonferroni tests for pairwise comparisons, p < 0.001 (significant when comparing no appendicitis with uncomplicated appendicitis, and uncomplicated appendicitis with complicated appendicitis). p < 0.05 was considered statistically significant

There were no significant differences in serum LRG1 concentrations between the groups. The median urine LRG1 concentration in the uncomplicated appendicitis group was 0.10 (IQR 0.02–0.29) μg/mL, which is significantly lower than in the no appendicitis group (0.33 [0.10–0.72] μg/mL) and in the complicated appendicitis group (0.29 [0.12–1.07] μg/mL), p < 0.001 (Table 1, Fig. 2a and b).

### Effects on odds of all cases of appendicitis and cases of complicated appendicitis

Among the demographic variables, male gender was significantly associated with increased odds of appendicitis. A long symptom duration (> 96 h) was significantly associated with decreased odds of appendicitis, compared to a symptom duration of < 24 h. Increased levels of all standard inflammatory markers (CRP, leukocytes, neutrophils, and neutrophil percentages) were significantly associated with increased odds of appendicitis. Notably, higher serum LRG1 concentrations were inversely associated with appendicitis (OR 0.96 [95% CI 0.93–0.99], p = 0.008. This association remained after adjustment for age, sex, and symptom duration (aOR 0.95 [95% CI 0.92–0.98], p = 0.003). Urine LRG1 concentration was not significantly associated with the risk of appendicitis (Tables 2 and 3).

**Table 2:** Unadjusted associations between independent variables and the odds for appendicitis in 172 children with suspected appendicitis

	No appendicitis n = 40	Appendicitis n = 132	OR (95% CI)	p value
Age (years)	11 (9–12.75)	10 (8–12)	0.95 (0.85–1.07)	0.426
Sex (male)	16 (40%)	82 (62%)	2.46 (1.19–5.07)	**0.015**
Symptom duration (h)				
0–24	14 (35%)	58 (44%)	Ref	Ref
24–48	13 (33%)	49 (37%)	0.91 (0.39–2.12)	0.827
48–96	9 (23%)	21 (16%)	0.56 (0.21–1.49)	0.248
> 96	4 (10%)	2 (2%)	0.12 (0.02–0.73)	**0.021**
CRP (mg/L)	12 (5–41)	31 (13–72)	1.01 (1.00–1.02)	**0.042**
Leukocytes (× 10^9^/L)	10.0 (6.7–14.8)	15.1 (12.3–18.7)	1.17 (1.08–1.27)	** < 0.001**
Neutrophils (× 10^9^/L)	6.5 (4.2–10.6)	12.2 (9.6–15.4)	1.21 (1.11–1.33)	** < 0.001**
Neutrophil percentage (%)	67 (57–77)	81 (76–86)	1.07 (1.03–1.10)	** < 0.001**
AIR score	4 (3–6)	7 (5–9)	1.43 (1.22–1.68)	** < 0.001**
Appendicolith	0 (0%)	33 (25%)	N/A	N/A
Serum LRG1 (μg/mL)	27.6 (19.7–36.3)	24.3 (18.8–30.1)	0.96 (0.93–0.99)	**0.008**
Urine LRG1 (μg/mL)	0.33 (0.10–0.72)	0.16 (0.05–0.46)	0.99 (0.89–1.08)	0.759

Values presented as median (IQR) and as absolute number and percentage of patients; n (%). Univariate logistic regression was used, with results presented as Odds Ratios (ORs) with 95% confidence intervals (CIs)

p < 0.05 was considered statistically significant (in bold)

Symptom duration, CRP, and leukocytes n = 40 and 130; neutrophils and neutrophil percentage n = 39 and 129, due to missing values

*CRP* C-reactive protein, *AIR score* Appendicitis Inflammatory Response score, *LRG1* Leucine-rich alpha-2 glycoprotein 1, *N/A* Not applicable

**Table 3:** Adjusted associations between serum and urine LRG and the odds of appendicitis in 172 children with suspected appendicitis

	No appendicitis	Appendicitis	aOR (95% CI)	p value
Serum LRG1 (μg/mL)			0.95 (0.92–0.98)	**0.003**
Urine LRG1 (μg/mL)			1.02 (0.91–1.15)	0.719

Multivariable logistic regression with adjustment for age, sex and symptom duration was used, with results presented as adjusted Odds Ratios (aORs) with 95% confidence intervals (CIs)

p < 0.05 was considered statistically significant (in bold)

When only analyzing the children with confirmed appendicitis and looking at the effects of the different clinical and laboratory variables on the odds of complicated appendicitis, young age was significantly associated with increased odds. So was a symptom duration of 48–96 h, compared to < 24 h. Higher CRP was also significantly associated with increased odds of a complicated disease course, while leukocytes, neutrophils and neutrophil percentages were not. Additionally, higher AIR scores and the presence of an appendicolith were also associated with increased odds of complicated appendicitis. Neither serum not urine LRG1 concentrations were significantly associated with the odds of complicated appendicitis (Tables 4 and 5).

**Table 4:** Unadjusted independent variables for complicated appendicitis in 132 children with appendicitis

	Uncomplicated appendicitis n = 76	Complicated appendicitis n = 56	OR (95% CI)	p-value
Age (years)	11 (9–13)	9 (7–11.75)	0.85 (0.75–0.95)	**0.006**
Sex (male)	46 (61%)	36 (64%)	1.18 (0.58–2.40)	0.660
Symptom duration (h)				
0–24	40 (53%)	18 (33%)	Ref	Ref
24–48	27 (36%)	22 (40%)	1.81 (0.82–4.00)	0.141
48–96	8 (11%)	13 (24%)	3.61 (1.27–10.23)	**0.016**
> 96	0 (0%)	2 (4%)	N/A	N/A
CRP (mg/L)	21 (7.7–39)	51 (27–121)	1.02 (1.01–1.02)	** < 0.001**
Leukocytes (× 10^9^/L)	14.4 (11.8–18.0)	15.8 (13.0–19.0)	1.06 (0.98–1.14)	0.128
Neutrophils (× 10^9^/L)	11.6 (9.3–14.7)	12.9 (10.3–15.8)	1.06 (0.98–1.15)	0.128
Neutrophil percentage (%)	80 (75–85)	83 (76–86)	1.04 (0.99–1.08)	0.078
AIR score	6 (5–8)	8 (6.25–10)	1.40 (1.18–1.66)	** < 0.001**
Appendicolith	13 (17%)	20 (36%)	2.67 (1.18–6.04)	**0.018**
Serum LRG1 (μg/mL)	24.8 (18.7–30.1)	23.4 (18.9–30.7)	0.99 (0.95–1.03)	0.557
Urine LRG1 (μg/mL)	0.10 (0.02–0.29)	0.29 (0.12–1.07)	1.17 (0.98–1.40)	0.089

Values presented as median (IQR) and as absolute number and percentage of patients; n (%). Univariate logistic regression was used, with results presented as Odds Ratios (ORs) with 95% confidence intervals (CIs)

p < 0.05 was considered statistically significant (in bold)

Symptom duration, CRP, leukocytes n = 75 and n = 55, and neutrophils, and neutrophil percentages n = 74 and 55, due to missing values

*CRP* C-reactive protein, *AIR score* Appendicitis Inflammatory Response score, *LRG1* Leucine-rich alpha-2 glycoprotein 1, *N/A* Not applicable

**Table 5:** Adjusted associations between serum and urine LRG with the odds of complicated appendicitis in 132 children with appendicitis

	Uncomplicated appendicitis	Complicated appendicitis	aOR (95% CI)	p value
Serum LRG1 (μg/mL)			0.99 (0.95–1.04)	0.778
Urine LRG1 (μg/mL)			1.18 (0.97–1.43)	0.107

Multivariable logistic regression with adjustment for age, sex and symptom duration was used, with results presented as adjusted Odds Ratios (aORs) with 95% confidence intervals (CIs)

p < 0.05 was considered statistically significant

### Diagnostic and discriminative performance of serum and urine LRG1 compared to standard inflammatory markers

**Fig. 3:**
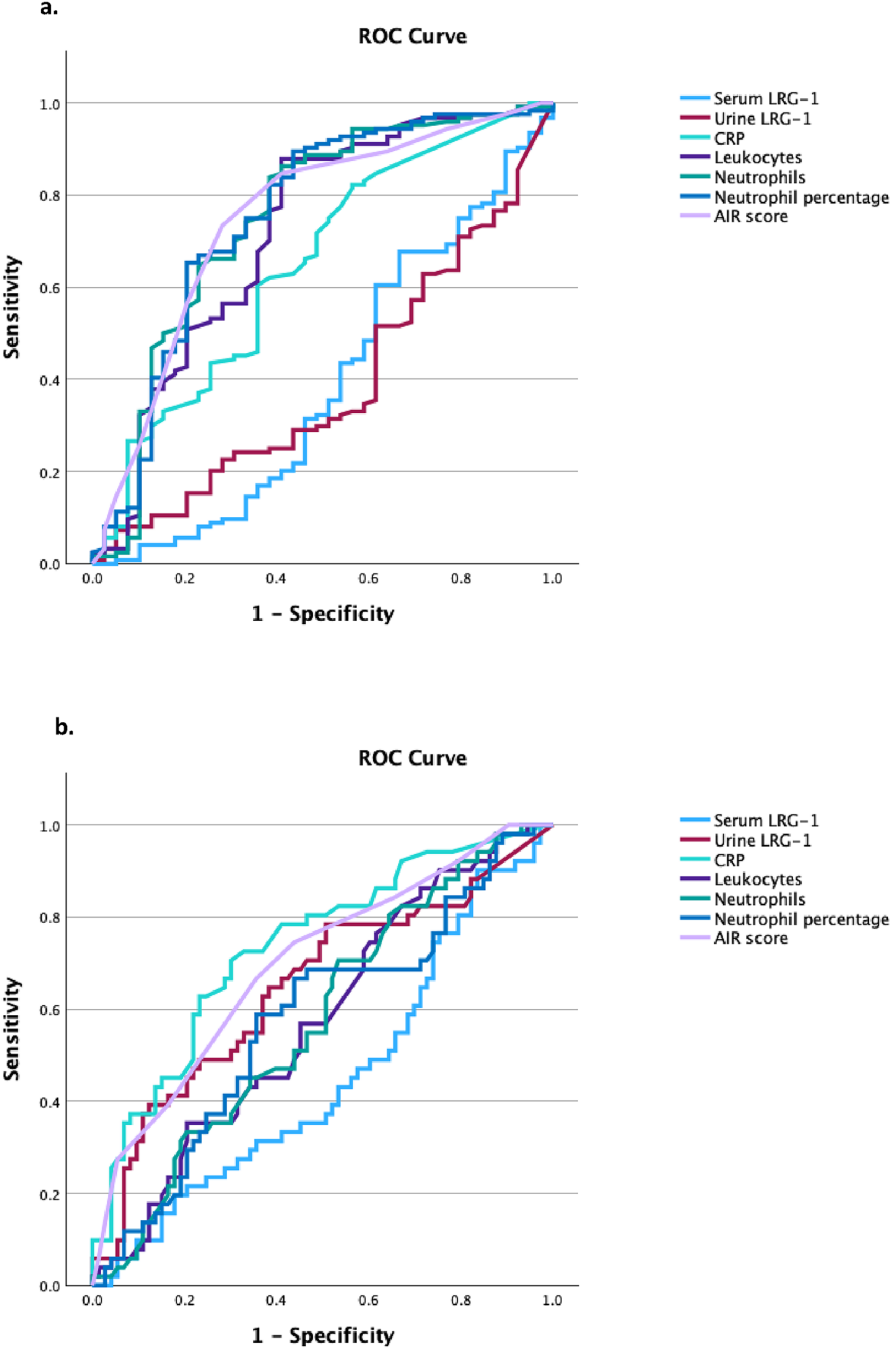
**a** Diagnostic performance of serum and urine LRG1 compared to standard laboratory tests as well as AIR score, to diagnose appendicitis in 172 children with suspected appendicitis. **b** Diagnostic performance of serum and urine LRG1 compared to standard laboratory tests as well as AIR score, to diagnose complicated appendicitis in 132 children with appendicitis

In the ROC curves for all cases of appendicitis, including all children with suspected appendicitis, both serum and urine LRG1 have AUCs of 0.39 (95% CI 0.28–0.50) and 0.39 (0.29–0.49), respectively. Neutrophils, neutrophil percentage and AIR score received the highest AUCs at 0.75 (Fig. 3a). Among all inflammatory markers, urine LRG showed the highest specificity of 95% (95% CI 83–99), and a PPV of 83% (53–96) (Table 6).

**Table 6:** Diagnostic, predictive, and ROC AUC values for serum and urine LRG1 as well as standard inflammatory markers and AIR score for **a** appendicitis in 172 children with suspected appendicitis, **b** complicated appendicitis 132 children with appendicitis

a. 172 children with suspected appendicitis					
	Sensitivity	Specificity	PPV	NPV	ROC AUC
Serum LRG1 (20.01 μg/mL)	69 (60–77)	33 (19–49)	77 (73–81)	24 (16–35)	0.39 (0.28–0.50)
Urine LRG1 (3.34 μg/mL)	8 (4–14)	95 (83–99)	83 (53–96)	24 (22–25)	0.39 (0.29–0.49)
CRP (9.35 mg/L)	83 (76–89)	45 (29–62)	83 (79–88)	45 (33–58)	0.65 (0.55–0.75)
Leukocytes (10.40 × 10^9^/L)	89 (82–93)	58 (41–73)	87 (82–91)	61 (47–73)	0.72 (0.62–0.82)
Neutrophils (8.65 × 10^9^/L)	85 (77–90)	62 (45–77)	88 (83–92)	55 (43–66)	0.75 (0.65–0.85)
Neutrophil percentage (70.64%)	88 (82–93)	58 (41–73)	87 (82–91)	61 (47–73)	0.75 (0.66–0.85)
AIR score low 0–3 points	89 (83–94)	36 (21–53)	82 (78–85)	52 (36–68)	0.75 (0.66–0.84)
AIR score high 9–12 points	26 (19–35)	90 (76–97)	89 (76–96)	28 (25–31)	

b. 132 children with appendicitis					
	Sensitivity	Specificity	PPV	NPV	ROC AUC
Serum LRG1 (15.70 μg/mL)	91 (81–97)	17 (9–27)	45 (42–48)	72 (50–87)	0.45 (0.35–0.55)
Urine LRG1 (0.10 μg/mL)	80 (66–90)	51 (40–63)	55 (48–61)	78 (67–86)	0.65 (0.55–0.75)
CRP (31.50 mg/L)	71 (57–82)	69 (58–79)	63 (54–71)	76 (68–83)	0.74 (0.65–0.83)
Leukocytes (12.35 × 10^9^/L)	84 (71–92)	33 (23–45)	48 (43–53)	74 (59–85)	0.58 (0.48–0.68)
Neutrophils (11.00 × 10^9^/L)	71 (57–82)	47 (36–59)	50 (43–57)	69 (58–78)	0.58 (0.48–0.68)
Neutrophil percentage (81.70%)	58 (44–71)	65 (53–76)	55 (46–64)	68 (60–75)	0.58 (0.47–0.68)
AIR score low 0–3 points	96 (87–100)	15 (8–25)	45 (42–47)	85 (56–96)	0.70 (0.61–0.79)
AIR score high 9–12 points	40 (27–55)	84 (73–91)	64 (49–76)	66 (61–72)	

Values presented as % and Area Under the Curve (AUC) scores from Receiver Operating Characteristics (ROC) analyses, with 95% Confidence Intervals (CIs)

*LRG1* Leucine-rich alpha-2 glycoprotein 1, *CRP* C-reactive protein, *AIR score* Appendicitis Inflammatory Response score, *PPV* Positive Predictive Value, *NPV* Negative Predictive Value

When it comes to identifying the children with complicated disease among the 133 with appendicitis, the AUC for serum LRG1 was quite close to 0.5 at 0.45 (95% CI 0.35–0.55), and for urine LRG1 it was 0.65 (0.55–0.75). CRP had the highest AUC at 0.74 (0.65–0.83), followed by AIR score at 0.70 (0.61–0.79) (Fig. 3b and Table 6).

## Discussion

The quest for better and safer diagnostic tools for appendicitis and complicated appendicitis in children is surely a great task, engaging many researchers and clinicians worldwide. To date, inflammatory blood markers as well as imaging studies such as ultrasonography or are often utilized in addition to clinical assessment of a child with acute abdominal pain [20, 21]. However, the diagnostic accuracies of standard inflammatory markers vary [20], and imaging studies are not always available and may be user dependent [22]. The present study sheds some new and important light on the diagnostic utility of serum and urine LRG1. Our results indicate that urine LRG1 might have the potential to differentiate between uncomplicated and complicated appendicitis in children, but with a lesser accuracy than currently more readily available diagnostic aids such as CRP and AIR score. Additionally, urine LRG had a high specificity and PPV for diagnosing appendicitis among all children with suspected appendicitis. Serum LRG1, on the contrary, does not seem to have a role in the assessment of a pediatric patient with acute abdominal pain.

To our knowledge, only 4 previous studies have investigated the diagnostic performance of serum LRG1 for pediatric appendicitis [10, 23–25]. All but one [23] of these studies have control groups consisting of patients with non-appendicitis acute abdominal pain, much like in our study. Still, their results indicate a good diagnostic performance of serum LRG1, especially one in which there was no overlap between serum LRG1 concentrations in the patients with and without appendicitis, generating an astonishing ROC AUC of 1.0 [24]. However, when evaluated on a small adult population (n = 28), serum LRG1 did not differ between the ones with and without appendicitis [26]. We can only speculate on why our results are fundamentally different from previous studies on pediatric patients. First, we must consider the study population. In our cohort, an absolute majority of the patients (78%) were diagnosed with appendicitis – a much greater proportion than in the previous studies [12]. This indicates that the threshold for referral of patients from either a pediatrician or a nurse at the pediatric ED, or from a family practician, to the on-call pediatric surgeon due to suspected appendicitis was quite high. And hence, even among the patients in the non-appendicitis group, the suspicion of appendicitis was high, even though most of them were diagnosed with unspecified abdominal pain. Worthy of mentioning is that the digital medical record system at our hospital includes all hospitals in the region, and that we therefor are certain that none of the patients sent home were diagnosed with appendicitis during the weeks after inclusion in the study, at least not in the region. This does not, however, exclude the possibility of spontaneously resolving appendicitis among these patients, who in that case would have been misclassified.

Another possible explanation for the vastly divergent results of different studies on LRG1 and appendicitis is the large selection of commercially available test kits used [12]. To our knowledge, the ELISA test kit used in this study (Cusabio, Hubei Province, China, CSB-E12962 h) has only been used in one previous appendicitis study – namely one from our group [14]. This previous study showed promising diagnostic properties of urine LRG1, especially when combined with urine creatinine. Although the study population of our previous study was substantially smaller than the current one, this indicates that the use of different test kits alone cannot entirely explain the different results.

In our cohort, urine LRG1 had a high specificity and PPV for diagnosing appendicitis among all children with suspected appendicitis. Unfortunately, the sensitivity and NPV in this setting were disappointingly low. Urine LRG1s ability to correctly identify the patients with complicated disease was better than both leukocytes, neutrophils and percentage neutrophils, but poorer than CRP and AIR score. Still, even though an AUC over 0.5 is indicative of some discriminative capacity, generally only values above 0.7 are considered acceptable [27]. The rationale behind our three AUC values below 0.5, is that the median concentrations of serum and urine LRG1 in the appendicitis were lower than in the non-appendicitis, and that the median concentration of serum LRG1 was lower in the complicated appendicitis coup compared to the uncomplicated appendicitis group, which is also reflected in the negative associations from the logistic regression analyses. Based on the results from previous studies indicating a positive association between increased LRG1 concentrations and appendicitis, the test was set up in a similar matter and not inversed.

Only a few previous studies have evaluated both the diagnostic and discriminative performances of urine LRG1. Kakar et al. concluded that the diagnostic performance of urine LRG1 (AUC 0.7) were better than its discriminative performance (AUC 0.6) [23]. In this study, however, the non-appendicitis group was comprised of patients who did not have acute abdominal pain, but instead different traumatic and non-inflammatory conditions – a setting rather far from the clinical one. Kharbanda et al. found that urine LRG1 in was significantly higher among pediatric patients with perforated appendicitis compared to those with non-perforated appendicitis. Unfortunately, they do not seem to have investigated the discriminative performance of LRG1 in this setting further. They did however conclude that leukocytes performed better than serum and urine LRG1 in diagnosing appendicitis [10].

As previously mentioned, only a small proportion of the included patients had non-appendicitis abdominal pain, and the predictive values of the different biomarkers need to be interpreted in the light of the high appendicitis prevalence. One must also consider if our current study population accurately reflects the intended target population for LRG1 utility. The results of our study do not support an introduction of LRG1 in clinical practice to help differentiate between uncomplicated and complicated appendicitis, since both CRP and AIR scored showed better discriminative performances. Furthermore, one could argue the great advantage of urine LRG1 being non-invasive is somewhat lost if the patient must go through venipuncture anyhow, for example to obtain preoperative blood tests. And in the hospital setting, further evaluations such as ultrasonography are often carried out. Apart from being diagnostic, ultrasonography can also identify appendicoliths, which are associated with increased risk of complicated appendicitis [28]. It is therefore possible that urine LRG1 could be best used to rule out appendicitis in patients with a low pretest probability, and evaluation of the biomarker in this setting, for example at a primary care facility, would be of great interest.

The main strength of the study lies in the prospective study design, with standardized collection of data and clear definitions of appendicitis and its’ different severities. Another strength is that the cohort is relatively substantial compared to previous studies on LRG1. Of course, the results of the current study must be interpreted in the light of its limitations. The first one includes the quite high number (n = 43) of excluded patients, for which we don’t have demographic data. This introduces the potential of selection bias. Secondly, we did not analyze urine creatinine and were therefore unfortunately not able to adjust our results for possible dehydration – something that has been shown to increase the diagnostic performance of urine LRG1 in previous studies [14].

## Conclusion

Urine LRG1 might be utilized in children both to diagnose appendicitis and to differentiate between uncomplicated and complicated appendicitis. However, it’s discriminative performance is poorer than the ones for CRP and AIR score. The clinical value of serum LRG1 seems limited since it was outperformed by all other included inflammatory markers, and in contrast to previous studies our results show an inverse association between serum LRG1 and the odds of appendicitis. The diagnostic utility of urine LRG1 should be evaluated in a different setting, since it probably could come to best use in patients with a low pretest probability for appendicitis, in order to rule out the diagnosis and send the patient home without any blood sampling.

## Data Availability

No datasets were generated or analysed during the current study.
